# Hand Hygiene Evaluation Using Two Different Evaluation Tools and Hand Contamination of Veterinary Healthcare Workers in a Swiss Companion Animal Clinic

**DOI:** 10.3390/vetsci8110260

**Published:** 2021-11-02

**Authors:** Kira Schmitt, Anna Barbara Emilia Zimmermann, Roger Stephan, Barbara Willi

**Affiliations:** 1 Section of Veterinary Bacteriology, Institute for Food Safety and Hygiene, University of Zurich, Winterthurerstrasse 272, CH-8057 Zurich, Switzerland; kira.schmitt@uzh.ch (K.S.); annabarbaraemilia.zimmermann@uzh.ch (A.B.E.Z.); roger.stephan@uzh.ch (R.S.); 2 Graduate School for Cellular and Biomedical Sciences, University of Bern, Mittelstrasse 43, CH-3012 Bern, Switzerland; 3 Clinic for Small Animal Internal Medicine, University of Zurich, Winterthurerstrasse 260, CH-8057 Zurich, Switzerland

**Keywords:** hand, hygiene, infection prevention control, hospital, antibiotic resistance

## Abstract

Hand hygiene (HH) is the most important measure to prevent nosocomial infections. HH compliance in companion animal clinics has been reported to be poor. The present study compared an online application with the WHO evaluation form to assess the WHO five moments of HH in a Swiss companion animal clinic. In 202 hand swabs from 87 staff members, total viable count (TVC) before and after patient contact was evaluated and the swabs were tested for selected antimicrobial resistant microorganisms of public health importance. HH compliance (95% confidence interval) was 36.6% (33.8–39.5%) and was similar when assessed with the two evaluation tools. HH differed between hospital areas (*p* = 0.0035) and HH indications (*p* < 0.0001). Gloves were worn in 22.0% (18.0–26.6%) of HH observations and were indicated in 37.2% (27.3–48.3%) of these observations. Mean TVC before patient contact was lower (0.52 log CFU/cm^2^) than after patient contact (1.02 log CFU/cm^2^) but was similar before patient contact on gloved and ungloved hands. Three hand swabs (1.5% (0.4–4.3%)) were positive for methicillin-resistant *Staphylococcus aureus.* Gloving should not be regarded as a substitute for HH. Overall, HH in companion animal medicine should urgently be fostered.

## 1. Introduction

Hand hygiene (HH) in companion animal clinics is of particular importance as intensive medical care of dogs and cats can be associated with nosocomial infections. HH is particularly crucial for geriatric and immunosuppressed animal patients which are more susceptible to infections and subsequent severe negative health outcomes [1,2,3,4]. Companion animals often receive antimicrobial therapy, including high priority critically important antimicrobials and last resort antibiotics, such as carbapenems [5,6,7,8,9,10,11,12,13,14,15,16]. Infection prevention and control standards in the companion animal healthcare sector are often insufficient and can contribute to the distribution of antimicrobial resistant microorganisms (ARM) between the environment and healthcare personnel [17]. Additionally, the close contact between owners and their pets poses the risk of transmission of ARM, such as extended-spectrum beta-lactamase-producing Enterobacteriaceae (ESBL-E) [18,19,20,21]. 

HH is the most important prevention measure against nosocomial infections in human medicine [22,23,24]. Approximately 30% of hospital-acquired infections are considered preventable [25]. Healthcare workers’ hands are one of the main risk factors for transmission of microorganisms between patients and the environment [26]. In a recent systematic review, average baseline HH compliance among healthcare workers in human adult intensive care units was reported to be 51.5%, increasing to 80.1% after intervention [27].

The WHO defines five indications (moments) in which HH should be carried out. They represent those situations which carry the highest risk for contamination of medical personnel’s hands inadvertently leading to transmission of pathogens [26,28]. Direct observation of staff during patient care by trained and validated observers using online tools or observation forms is considered the gold standard for monitoring hand hygiene [26]. HH is defined as the use of hand disinfection with an alcohol-based disinfectant or the washing of hands with soap and water. Alcohol-based hand disinfectant should contain 60–95% ethanol or isopropyl alcohol [29]. When indicated, for example, when hands are visibly soiled or dirty, hands should be washed with a nonmedicated soap. For optimal compliance, hand hygiene products should be readily available [30]. The use of gloves is disregarded as HH and has frequently been discussed as a barrier to HH, since gloves do not provide complete protection against hand contamination. Pathogens can be transferred onto the hands through small defects in the gloves or through contamination of the hands during glove removal. The WHO recommends the use of gloves to be limited to contact with potentially infectious material or patients, expected body fluid exposure risk, for clean/aseptic/invasive procedures and for cleaning and disinfection. They should only be worn for as long as necessary and contact with “clean” surfaces should be avoided [26,30]. HH should also be practiced before putting on gloves and after glove removal in accordance with the WHO five moments of HH, independent of glove usage.

HH compliance in companion animal medicine has been assessed in few studies. Compliance ranged from 14% to 27% before intervention, and up to 14% to 42% after intervention [31,32,33]. These results are in line with a recent study from Switzerland, which evaluated HH compliance in seven companion animal clinics and practices. Adherence of employees to the WHO five moments of HH was insufficient in all institutions and ranged from 26% to 47% [34]. HH compliance did not differ between large and medium-sized clinics or between the three large clinics included in the study. However, HH compliance was significantly different between the five HH indications and between the clinical areas in the large clinics [34].

Overall, data regarding HH compliance in companion animal medicine, especially regarding the use of gloves and the type of HH carried out, is limited. Furthermore, no data is available on hand swabs in conjunction with HH evaluation. The goal of this study was to assess the WHO five moments for HH, the type of HH being implemented, and the use of gloves in a tertiary care facility in Switzerland, using the online application CleanHands (Swissnoso, National Centre for Infection Prevention, Bern, Switzerland) and the WHO evaluation form [35]. Additionally, the present study investigated hand swabs of companion animal healthcare workers for contamination with ESBL-E, carbapenemase-producing Enterobacteriaceae (CPE), vancomycin-resistant enterococci (VRE), methicillin-resistant *Staphylococcus aureus* (MRSA), and *Staphylococcus pseudintermedius* (MRSP). Hand contamination was evaluated in relation to HH procedures in different clinical areas in the care facility.

## 2. Materials and Methods

This study was conducted at a large tertiary referral hospital for small animals, covering a wide range of specialty services, with approximately 200 employees and 10,000 patients per year offering a 24-h, 365 days a year emergency service including an intensive care unit (ICU). At the beginning of the study, an information sheet was distributed by the clinic’s communication platform to announce the study to the clinical staff. The clinic used the same alcohol-based hand disinfectant (Desmanol^®^ pure, 75% Propan-2-ol, Schülke & Mayr AG, Zurich, Switzerland) and soap (S&M^®^ wash lotion, Schülke & Mayr AG, Zürich, Switzerland) in all clinical areas and the products remained unchanged throughout the study period.

### 2.1. Hand Hygiene Evaluation

HH compliance was evaluated according to the WHO five moments of HH (after body fluid exposure risk, after patient contact, after touching the patient’s surroundings, before clean/aseptic/invasive procedures, and before patient contact; Table S1) as previously described [34], using the CleanHands application (Swissnoso, National Centre for Infection Prevention) [36] as well as the WHO evaluation form [35]. Both tools were used to equally evaluate HH (i.e., carried out or not) in five different clinical areas: consultation rooms, wards, examination area for hospitalized patients, intensive-care unit (ICU) and pre-operation preparation area. Additionally, HH compliance across professional groups (veterinarians; nurses and others, i.e., personnel not allocated to the aforementioned categories, such as students and technicians) was analyzed. All observations were carried out by the same observer over a period of ten weeks. The observer had been previously trained by an experienced observer [34]. For ethical reasons, and in accordance with the WHO recommendations, the observer introduced herself and stated the reason for her presence before the start of each session without going into more detail about the indications or HH activities [30]. The observer then positioned herself inconspicuously and started the session. To minimize selection bias, each session was started in a randomly selected clinical area. After 30 min of observation, the evaluation was continued in the following order in another area: ICU, wards, consultation rooms, examination area for hospitalized patients, and finally the pre-operation preparation area. If there was no activity in a particular area for more than five minutes, the evaluation was continued in a different area. The first person to be encountered in the respective clinical area was observed during an entire activity. Another randomly selected person was observed thereafter. No more than three people were observed at once [36,37] and a maximum of two activities per session were observed per healthcare worker. If more than one indication of hand hygiene was given, the following prioritization was applied: before clean/aseptic/invasive procedure, after body fluid exposure risk, after patient contact, before patient contact, and after touching the patient’s surroundings. The prioritization is based on a local consensus, weighing the perceived risk of infection for the patient against the risk of microbiological contamination of the healthcare workers’ hands. The prioritization scheme has been used in previous studies in human and veterinary medicine [26,34]. In contrast to the CleanHands application available at the time of this study, the WHO evaluation form additionally allows recording of the type of HH that was practiced (hand disinfection with alcohol-based hand rub or hand washing with water and soap) and documenting the use of gloves and whether HH was carried out according to WHO criteria when using gloves. Glove usage was categorized as appropriate according to WHO guidelines when activities involved expected body fluid exposure risk and when clean/aseptic/invasive procedures were performed. HH and the use of gloves was not evaluated during contact precautions when treating infectious patients, as the clinic had defined specific hygiene measures for these instances. After digital recording of the observations with the CleanHands application, the data was extracted from the software as Excel files for further statistical analyses. The data obtained with the WHO evaluation tool was manually transferred to Excel files for statistical analyses.

### 2.2. Hand Swabs Collection 

Hand swabs of the entire dominant hand palm, fingers and thumb were collected before and after patient contact using a sterile cotton swab moisturized with 0.85% saline solution. Hand swabs were processed immediately after sample collection. If gloves were worn, hand swabs were taken from the gloved hand before and after patient contact. The same type of gloves (Sempercare^®^ velvet, non-sterile, powder-free single use nitrile examination and protective gloves, IVF Hartmann AG, Neuhausen, Switzerland) were available in all areas of the clinic and remained the same throughout the study period. To reduce potential observer bias, hand swabs were taken during busy daily procedures and in areas with a high density of patients and personnel (wards, 62 samples; examination area, 66 samples; ICU, 74 samples, respectively). The healthcare workers were approached immediately before animal contact without any prior announcement. A coded sample collection procedure was used and hence no personal data was collected from the study participants to ensure that employers did not feel obliged to change their individual hand hygiene behavior. The hand swab collection and the aforementioned hand hygiene observations were conducted in a mutually exclusive fashion. For data interpretation purposes, the hand hygiene procedure carried out before and after hand swab collection was noted. All study participants gave written informed consent. This study was approved by the Swiss Ethics Committee on research involving humans (approval No. 2019-00768).

### 2.3. Microbiological Analysis

Hand swabs were homogenized for 60 s in 10 mL peptone water (BioRad, Hercules, CA, USA) using a Stomacher^®^ 400 (Seward, Worthing, UK). Afterwards, 1 mL was used to prepare decimal dilution series (0.85% saline solution). Aliquots of 0.1 mL were then transferred to plate count agar (BioRad, Hercules, CA, USA) and distributed by applying the spreading method. Total viable counts (TVC) were calculated as CFU/cm^2^ after incubation for 72 h at 30 °C. TVC were expressed as log CFU/cm^2^. The definitions for hand surface area and the calculation methods vary across the literature and depend on the investigated population. The size of the hand was determined as 100 cm^2^ for the purpose of this study [38,39]. The detection limit was 1 CFU/cm^2^. A log value of zero was used for counts below the detection limit. 

The remaining homogenate of each hand swab sample was enriched (37 °C, 24 h), followed by selective enrichment for ESBL-E and CPE in Enterobacteriaceae enrichment broth (Oxoid, Hampshire, UK), in BHI (BioRad, Hercules, CA, USA) with 6.5% saline solution for VRE, and additionally in Mueller Hinton broth (Oxoid, Hampshire, UK) with 6.5% saline solution followed by an enrichment in tryptone soy broth (Becton Dickinson, Allschwil, Switzerland) with 4 mg/L cefoxitin and 75 mg/L aztreonam for the detection of MRSA and MRSP. ESBL-E were screened using chromogenic medium Brilliance™ ESBL Agar (Oxoid, Hampshire, UK), CPE by using chromID^®^ CARBA SMART Bi-Plate-Agar (bioMérieux, Marcy-l’Étoile, France), VRE by using Brilliance™ VRE Agar (Oxoid, Hampshire, UK) and MRSA and MRSP by using Brilliance™ MRSA2 Agar (Oxoid, Hampshire, UK), according to the manufacturer’s instructions. Species identification was conducted by using a matrix-assisted laser desorption/ionization time-of-flight mass spectrometry (MALDI-TOF–MS, Bruker Daltronics, Bremen, Germany).

Polymerase chain reaction (PCR) was carried out to screen for the presence of genes encoding *bla*_CTX-M_ group enzymes, *bla*_SHV_ and *bla*_TEM_ as previously described [40,41,42,43]. PCR targeting *bla*_VIM_, *bla*_KPC_, *bla*_OXA-48_-like and *bla*_NDM_ genes was carried out using custom synthesized primers (Microsynth, Balgach, Switzerland) and conditions published previously [44,45]. Multiplex PCR for the presence of *vanA*, *vanB* and *vanC*_1,2,3_ was conducted as previously described using custom synthesized primers (Microsynth, Balgach, Switzerland) [46]. PCR for the presence of *mecA* and *mecC* was conducted using custom synthesized primers (Microsynth, Balgach, Switzerland) as previously described [47,48]. 

### 2.4. Statistical Analysis

For statistical analysis, commercially available GraphPad PRISM^®^ software (San Diego, CA, USA) was used. Descriptive statistics were conducted for HH compliance (%, number of correct HH events per total number of observed HH events) and the TVC on the hand swabs. Binominal confidence intervals for HH compliance and glove usage were calculated using the hybrid Wilson/Brown method [49]. Contingency tables were calculated using the chi-square test. For multiple comparisons of TVC between groups (before patient contact, after patient contact, gloves, no gloves), ordinary one-way ANOVA was carried out. Significance was set at *p* < 0.05.

## 3. Results

### 3.1. Hand Hygiene Compliance

Overall, 1165 HH evaluations were carried out: 810 of 1165 observations with the CleanHands application and 355 of 1165 with the WHO evaluation form. In 75 of 810 cases evaluated with the CleanHands application, HH could not be matched to any of the WHO five moments of HH, i.e., HH was carried out without any indication. These cases were therefore classified as “non-coded” and were left out of the statistical analysis, leaving a total of 1090 observations (Table 1). Overall, HH compliance (95% confidence interval) was 36.6% (33.8–39.5%). The observed HH compliance with the WHO observation form (34.1% (29.3–39.2%)) was not different to the one established with the CleanHands application (37.8% (34.4–41.4%)) (Table 1). Significant differences (*p* = 0.0035) were observed between different clinical areas (Table 1), with the highest compliance observed in the ICU (44.0% (37.9–50.3%)) and the lowest in the pre-operation preparation area (28.6% (22.9–35.0%)). HH was significantly (*p* < 0.0001) more commonly performed after body fluid exposure (55.8% (48.8–62.5%)) and after patient contact (51.6% (46.1–57.1%)) than after touching the patient’s surroundings (33.6% (25.4–43.0%)), before clean/aseptic/invasive procedures (14.3% (10.4–19.6%)) and before patient contact (23.5% (18.6–29.1%)) (Table 1). Differences within professional groups were not significant (*p* = 0.0879), but the group “others” (29.5% (23.4–36.5%)) tended to perform lower than veterinarians (38.5% (34.2–43.1%)) and nurses (37.6% (33.2–42.1%)) (Table 1).

### 3.2. Type of Hand Hygiene and Use of Gloves

The WHO observation form revealed that alcohol-based hand rub was used in 68 of 121 (56.2% (47.3–64.7%)) observations where HH was performed (n = 121), soap and water in 35 of 121 (28.9% (21.6–37.6%)) observations and both HH methods were applied in 18 of 121 (14.9% (9.6–22.3%)) observations. Gloves were worn in 78 of 355 (22.0% (18.0–26.6%)) observations. The use of gloves was indicated in 29 of these 78 (37.2% (27.3–48.3%)) observations. When using gloves, HH was carried out according to WHO recommendation in 18 of 78 (23.1% (15.1–33.6%)) observations.

### 3.3. Hand Swabs

**Figure 1:**
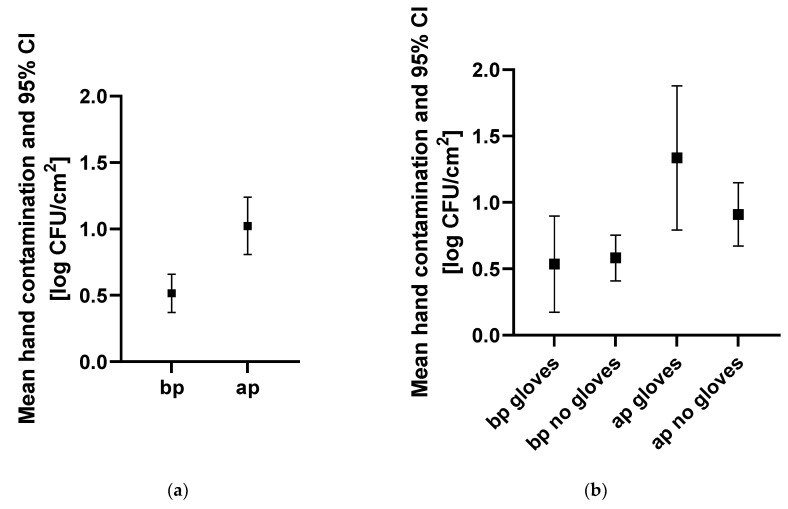

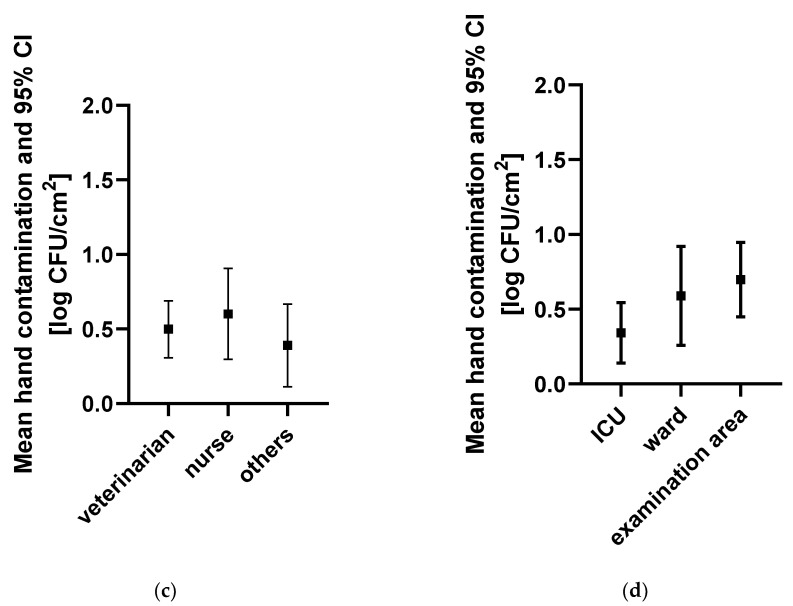
Hand contamination for different indications, professional groups, and clinical areas. Hand contamination (**a**) before and after patient contact, (**b**) before and after patient contact with and without gloves, (**c**) of different professional groups before patient contact, and (**d**) in different clinical areas before patient contact is indicated. Mean hand contamination and 95% CI are indicated in log CFU/cm^2^. Abbreviations: bp, before patient contact; ap, after patient contact; others, personnel not allocated to the categories, “veterinarian” or “nurse”, such as students and technicians; ICU, intensive care unit.

A total of 202 hand swabs (101 swabs before and 101 swabs after patient contact) were collected at 87 instances. Overall, mean TVC (95% confidence interval) before patient contact was lower (0.52 log CFU/cm^2^ [0.37–0.66]) than mean TVC after patient contact (1.02 log CFU/cm^2^ (0.81–1.24)) (Figure 1). When not wearing gloves, mean TVC before patient contact was lower (0.58 log CFU/cm^2^ (0.41–0.75)) than after patient contact (0.91 log CFU/cm^2^ (0.67–1.15)). Similarly, mean TVC on gloves was lower before (0.54 log CFU/cm^2^ (0.17–0.90)) than after patient contact (1.34 log CFU/cm^2^ (0.79–1.88)). However, there was no difference in TVC before patient contact in staff wearing gloves versus not wearing gloves. Hand contamination was not significantly different (*p* = 0.1) between the areas. However, it was lowest in the ICU (0.34 log CFU/cm^2^ (0.14–0.54)) and highest in the patient examination area (0.70 log CFU/cm^2^ (0.45–0.95)). Additionally, there were no significant differences (*p* = 0.5774) in hand contamination between professional groups.

MRSA was isolated from three hand swabs (1.5% (0.4–4.3)). Two of these hand swabs were taken before patient contact and one after patient contact. All strains harbored the *mecA* gene. BZ_32_ap belonged to ST398 and spa type t011, BZ_38_bp to ST105 and spa type t002 and BZ_44_bp to ST 398 and spa type t011.

## 4. Discussion

Overall, HH compliance was poor (36.6%) in the companion animal clinic evaluated in this study. These results mirror those recently found in seven companion animal clinics and practices in Switzerland where the average HH compliance was 32%, ranging from 26% to 47% across the seven institutions. The results are also in line with those from previous veterinary studies where compliance ranged from 14% to 27% before and 14% to 42% after intervention [31,32,33]. However, those studies did not evaluate HH compliance in accordance with the WHO criteria; hence, comparison is limited. Our results highlight the need to foster HH intervention and training in companion animal medicine, given that HH is amongst the most important prevention measures for nosocomial infections [25,50]. The findings are in contrast to results from studies conducted in human healthcare, where HH compliance is generally higher, although a wide range exists among these studies [27]. 

Both evaluation tools used in this study obtained comparable results. The method using the WHO observation form was more time consuming due to the manual extraction and analysis of the data. However, it allowed the evaluation of additional criteria such as the type of HH performed and the use of gloves. The recently updated CleanHands application (Swissnoso, National Centre for Infection Prevention) additionally records the latter but was unavailable at the time of this study [51].

The higher HH compliance in the ICU in comparison to the other clinical areas, combined with a low hand contamination, was surprising. Intensive-care environments such as the ICU or the pre-operation preparation areas have been previously associated with lower compliances due to a high “activity index”, i.e., number of observed HH opportunities per hour for each observation period [52] and higher-risk procedures [27,52,53]. Of note, the compliance levels observed in the ICU in this study were higher than those of a recent study conducted in seven companion animal clinics and practices across Switzerland [34]. In line with results from previous veterinary studies, HH compliance was highest after body fluid exposure and lowest before clean/aseptic/invasive procedures [31]. This implies that HH might mainly be used for self-protection instead of for patient protection [26].

Particular attention should be paid when using gloves, since wearing gloves is often misconceived as a substitute for HH [49,50,54,55]. In this study, gloves were worn in 22.0% of HH indications, but the use of gloves was, according to WHO standards, indicated in only 37.2% of these observations. Additionally, HH when wearing gloves was only conducted in 23.1% of the observations. In one study, observed HH compliance after glove removal was 39% [31]. Such low HH compliances are worrisome, as pre-existing defects, and damage during use and glove removal could pose potential hazards for hand contamination [53,54,55]. Therefore, wearing gloves is not considered a HH procedure by the WHO [26,30]. Of note, the TVC did not differ before patient contact in samples taken from gloved compared to ungloved hands of the healthcare workers. This underlines that wearing gloves does not automatically reduce the bacterial load on the hands and does not prevent transmission of pathogens. Moreover, gloves can potentially become contaminated when putting them on. Therefore, the HH indications apply regardless of the use of gloves [26,30]. 

Hands are regarded as the main risk factor for ARM transmission, and nosocomial pathogens have been reported on the hands of veterinary healthcare workers [56]. In this study, 1.5% of hand swabs tested positive for MRSA ST105 *spa* type t002 and ST398 *spa* type t011 harboring the *mecA* gene. MRSA ST105 is a frequently described ST, which has also been linked to an outbreak in a neonatal ICU [57]. The emergence of ST398 has been associated with infections in humans and animals, and has been isolated from a dog’s wound and from the nose of a veterinary staff member of the same clinic [58]. In dogs, unlike in humans, *Staphylococcus pseudintermedius*, not *Staphylococcus aureus*, has been described as the more prevalent opportunistic pathogen [59]. A systematic review analyzing fifty-nine articles comprising 6840 hand cultures determined a pooled prevalence of 4.3% for MRSA on the hands of healthcare workers in human medicine [60]. In veterinary medicine, most studies analyze nasal carriage, and only few determined contamination of hands. Studies have reported drug-resistant Enterobacteriaceae on the hands of veterinary staff [56] and nosocomial pathogens have been isolated from the hands of healthcare workers [61]. One study found a MRSA and MRSP prevalence of 7% and 2%, respectively, on the hands of veterinary staff [56], whereas another study found a prevalence of 0% for MRSA and 4.7% for MRSP [62]. However, comparison between studies is challenging, as the sampling methods, i.e., cotton swab, direct contact, glove juice method, and study protocols vary. 

The present study has some limitations. For one, the results of the HH observation could underly the Hawthorne effect, and thus be too optimistic, since direct observation could lead to a higher HH compliance [63,64]. However, according to WHO guidelines, direct observation is considered the gold standard when evaluating HH compliance [65]. Indeed, the WHO advises against hidden observation as it might be considered unethical to observe an individual’s behavior without informed consent. Hidden observation could also lead to mistrust amongst the employees. Furthermore, a proportion of the observations in this study were conducted in the consultation rooms, where an introduction to the animal owner was unavoidable. The Hawthorne Effect is, however, assumed to be transient and most pronounced at the beginning of the observation period. Therefore, the observation sessions were conducted over a ten-week period. An inconspicuous positioning of the observer, as in this study, can also further minimize observer bias. A second limitation is that the present study was conducted in only one clinic and extrapolation of these results to other companion animal clinics may not be relevant in all comparisons. Our results on hand hygiene compliance were, however, comparable to those of a previous study performed in seven companion animal veterinary institutions in Switzerland and should thus be applicable and may be considered relevant in a broader context [34]. Thirdly, the number of hand swabs was limited, considering the number of hand-patient contacts that took place during a day.

## 5. Conclusions

The present study found a largely insufficient HH compliance in a large veterinary referral clinic for companion animals in Switzerland. HH was often neglected before patient contacts and before clean/aseptic/invasive procedures. The use of gloves was common, and gloves were frequently worn without indication. TVC before patient contact was similar when wearing gloves versus when not wearing gloves, confirming that wearing gloves cannot be considered a HH procedure; a finding in line with the WHO recommendations. The online tool and the WHO observation form gathered comparable results. Although the WHO observation form is more time-consuming, it allows for additional evaluation aspects. Future online applications should allow to differentiate the HH procedure performed (water and soap and alcohol-based hand rub) and include the use of gloves. There is a need for evidence-based recommendations on hygiene intervention in veterinary care settings and training on adequate HH should be fostered.

## Figures and Tables

**Table 1:** Number of hand hygiene observations and hand hygiene compliance within clinical areas, professional groups, and hand hygiene indications.

	Total Number and Percentages of Hand Hygiene Observations	Hand Hygiene Compliance (95% CI)
Overall	1090	36.6% (33.8–39.5%)
CleanHands application	735 (67.4%)	37.8% (34.4–41.4%)
WHO evaluation form	355 (32.6%)	34.1% (29.3–39.2%)
Area		
Pre-operation preparation area	210 (19.3%)	28.6% (22.9–35.0%)
ICU	241 (22.1%)	44.0% (37.9–50.3%)
Consultation	194 (17.8%)	39.7% (33.1–46.7%)
Ward	215 (19.7%)	39.1% (32.8–45.7%)
Examination area	230 (21.1%)	31.3% (25.7–37.6%)
Professional group		
Veterinarian	457 (42.0%)	38.5% (34.2–43.1%)
Nurse	450 (41.2%)	37.6% (33.2–42.1%)
Others	183 (16.8%)	29.5% (23.4–36.5%)
Indication		
After body fluid exposure risk	199 (18.3%)	55.8% (48.8–62.5%)
After patient contact	314 (28.8%)	51.6% (46.1–57.1%)
After touching the patient’s surroundings	107 (9.8%)	33.6% (25.4–43.0%)
Before clean/aseptic/invasive procedure	223 (20.4%)	14.3% (10.4–19.6%)
Before patient contact	247 (22.7%)	23.5% (18.6–29.1%)

Abbreviations: ICU, intensive care unit; others, personnel not allocated to the categories, “veterinarian” or “nurse”, such as students and technicians.

## Data Availability

The datasets used and analyzed during the current study are available from the corresponding author upon reasonable request.

## Supplementary Materials

The following are available online at https://www.mdpi.com/article/10.3390/vetsci8110260/s1. Table S1: Indications for hand hygiene according to the WHO and examples.
