# Supplementary material for: Hand Hygiene Evaluation Using Two Different Evaluation Tools and Hand Contamination of Veterinary Healthcare Workers in a Swiss Companion Animal Clinic
Source: Vet Sci. 2021 Nov 2;8(11):260. doi: 10.3390/vetsci8110260 (PMC8623950; doi:10.3390/vetsci8110260)
Supplement: Supplementary file 1 [file vetsci-08-00260-s001.zip › vetsci-1297109-supplementary.pdf]

**Table S1. Indications for hand hygiene according to the WHO and examples.**
The indications and examples have recently been published[1]

| Indications for hand hygiene according to the WHO | Example |
|---|---|
| **After body fluid exposure risk** | After contact with urine, feces, blood, secretions, drainage, measuring of temperature |
| **After patient contact** | After clinical examination, after care of inpatients |
| **After patient surrounding** | After contact with the animal box, the transport box, the treatment table, the examination material, the infusion pump, ultrasound device (when being used), anesthesia device, treatment protocol, patient sheet, water/ feeding bowl |
| **Before clean/ aseptic/ invasive procedure** | Before taking blood, setting intravenous access, punctures, changing bandages, manipulating infusion tubes, intubation, parenteral administration of medication, administration of eye drops |
| **Before patient contact** | Before the clinical examination, before the care of inpatients |

[1]Reference: Schmidt JS, Hartnack S, Schuller S, Kuster SP, Willi B. Hand hygiene compliance in companion animal clinics and practices in Switzerland: An observational study. Vet Rec 2021: e307.
